# Simulation of the Nucleation and Crystal Growth Process in the Laser-Induced Deposition in Solution by a Lattice Boltzmann Method

**DOI:** 10.3390/nano12183213

**Published:** 2022-09-16

**Authors:** Yongsen He, Siyu Liu

**Affiliations:** School of Mechanical Engineering, Shanghai Jiao Tong University, Shanghai 200240, China

**Keywords:** laser-induced deposition in solution (LIDS), morphology control, process-condition-material relationship, pulsed laser, nucleation and crystal growth, lattice boltzmann method (LBM)

## Abstract

A Lattice Boltzmann model is proposed, combining the theories of nucleation and crystal growth for the study of the laser-induced deposition in solution (LIDS). The conjugate heat transfer and the natural convection of the liquid precursor were simulated with the evolving interface of crystal growth. In turn, the morphology of the deposited materials was affected by multiple process parameters, including conditions of chemical precursor and the laser-induced heat and mass transfer. Simulation results indicated that the morphology of deposited materials was mostly affected by the initial concentration of the precursor solution. Specifically, the nonuniformity of thin films was caused by the convection induced by the pulsed-laser, and the surface roughness was due to the competition of local structures for the precursor supply. A relationship of process-condition-material was established, providing guidance of choosing various parameters in LIDS for a desirable morphology of deposited material, facilitating the capabilities of pulsed lasers in precise control in nanomanufacturing.

## 1. Introduction

With the continuous development and progress of materials science, increasing attention has been paid to the new technologies of materials synthesis and nanomanufacturing. Hydrothermal methods are cost-effective and efficient liquid phase preparation technologies that have been developed rapidly in recent years, and in various fields, such as the piezoelectric, ferroelectric, ceramic powder, and oxide film fields [1,2,3]. Laser-induced chemical deposition in solution (LIDS), as an extension of the hydrothermal reaction, has become a promising approach in recent years [4,5]. In LIDS, a focused laser produced a micron-dimensional heated region, where the chemical reaction is confined. In contrast to conventional batch hydrothermal reactions, LIDS has great potential as a localized, single-step, and mask-less alternative in microelectronic processing. A wide range of nanomaterials and structures were deposited in high productivity [6,7,8].

Depositing materials with high quality is important for their application, such as in the microelectronics and display industry, the high conductivity of deposited metals is of the main interest [9]. Conductivity and other electrical properties are closely related to the thin film crystalline structure: single crystalline, polycrystalline or amorphous [10,11]. F the morphologies of deposited materials are determined by the process of nucleation and crystal growth. Therefore, understanding the dynamics associated with nucleation and the initial stage of thin film growth is important for controlling thin film properties [12].

Since there are a lot of variables that could be tuned during LIDS, it brings great potential for the precise control of the deposition reaction [13]. It was reported that a single laser pulse with proper energy could initiate and control the pathway of the nucleation of hematite nanocrystals [14]. During crystal growth, the peak power, average power, irradiation time, and so on will affect the morphology of deposited ZnO crystals [15]. However, it is still challenging to precisely manipulate heat and mass transfer only through experiments [11]. A process-condition-material relationship is necessary to be developed with the help of computational simulations [16,17]. Most simulations related to the laser applications use macroscopic methods such as FVM and FEM [18,19,20,21]. The multi-physics problems could be simulated with good predictions; however, the microstructure of deposited materials is usually difficult to capture. The lattice Boltzmann method (LBM), as a mesoscopic simulation method, has the superiority of incorporating temperature, concentration, and velocity field in a uniform formula diagram [22]. Chen Li [23,24] et al. investigated coupled multiple physicochemical thermal processes at the pore-scale. Mao Yudong [25] et al. used LBM employed to simulate the ultra-fast laser heating of a thin silicon film with nano-scale thickness; Alberto Cattenone [26] et al. used the lattice Boltzmann method to study multiphysics and phase transition during manufacturing; Z G Huang [27] et al. studied the material ejection from copper films induced by nanosecond laser pulses. Based on the above literature, although the deposition process is discussed using the LBM and the multi-physics coupling problem in the laser heating process is simulated numerically, the mutual effects of environmental conditions and deposited materials with a changing interface were not taken into account. Moreover, the precise control of the morphology of deposited materials were not discussed in depth. Furthermore, to the best of our knowledge, the conjugate heat transfer in LIDS and temperature field changes due to material deposition are rarely studied in the past literature.

In this study, we used the LBM combining with the nucleation and crystal growth model, to investigate the LIDS process, which involves multi-physics coupling simulation, conjugate heat transfer, and material deposition processes. Moreover, the model of nucleation and crystal growth led to an accurate simulation for the moving interface of the conjugate heat transfer. Here we studied the deposition of copper on silicon as an example case. Since Cu has superior conductivity and availability, it becomes the most attractive metal for on-chip metallization or integrated devices [28]. We simplified the deposition process as a precursor generation process, followed by a crystallization process, which includes nucleation and crystal growth. We used a stationary pulsed-laser beam to simplify the heating source for reaction, focusing on the investigation of morphology control, and hope to provide a reference for further laser applications [29,30], such as laser direct writing or scanning processes.

The paper is structured as follows: Section 2 describes the physical and chemical models and their basic assumptions; Section 3 introduces the numerical models, including the LB model for solving multi-field and its boundary conditions and the numerical model of the crystallization process; Section 4 analyzes the simulation results; Section 5 is the conclusion. Some mathematical details and more detailed results are collected in the Supplementary Information (SI).

## 2. Mathematical Model and Assumptions

### 2.1. The Thermal Model of Laser-Induced Deposition in Solution (LIDS)

**Figure 1:**
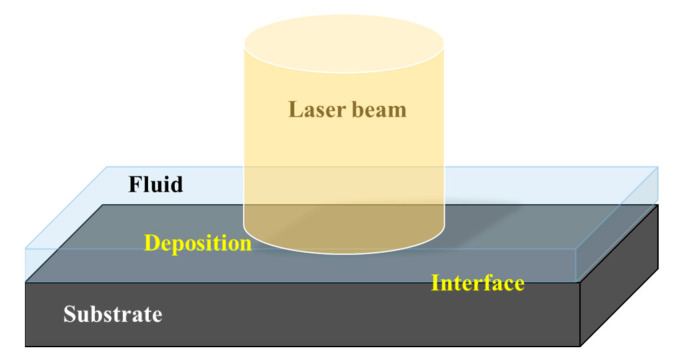
Schematic diagram of laser induced deposition.

The physical model of LIDS is shown in Figure 1. In a typical process of LIDS, the substrate absorbs the laser heating, leading to the temperature rising, and the local chemical reaction in the fluid is activated to generate the precursor of the deposits. Over time, when the precursor concentration in the solution exceeds the supersaturation level, it will heterogeneously nucleate and grow at the solid-liquid interface to form sediments.

The laser heating calculation area simulated in this paper includes fluid and solid area. The fluid-solid coupling heat transfer process and the liquid natural convection process are considered in the solution process, so it is a conjugate heat transfer process. In the calculation, the energy equations are the same for both regions. The incompressible N-S equation (NSE) and component transport equations are solved additionally in the fluid region. The governing equations could be solved by the following Equation (1).

(1) $$\frac{\partial \left(\rho \phi \right)}{\partial t}+div\left(\rho V\phi \right)=div\left(\Gamma_{\phi }grad\phi \right)+S_{\phi }$$

where: ρ is the general expression of density, V is velocity vector, ϕ is scalar, $\Gamma_{\phi }$ is general transport coefficient, $S_{\phi }$ is general source term. For different conservation equations, the parameters are as shown in the Table 1 Values of different governing equations Table 1.

The mean free path of molecules calculated by taking water on behalf of the aqueous solution is about $10^{-9}$m, and $L=4\times 10^{-3}$m the Kn number equal to$\;2.5\times 10^{-7}\ll 0.001$ [31]. Therefore, the continuum model used in this article is valid. The pulse laser width is 100 ns, which is much longer than the relaxation time of electron–electron collisions and electron–phonon interactions [32], each pulse irradiation was regarded as thermal equilibrium and the Fourier law was applied. For natural convection in this simulation, the Boussinesq assumption [33] is used, treating all the density in the solved equation as a constant value, except for the buoyancy term in the y-momentum Equation (2).

(2) $$F=\left(\rho -\rho_{0}\right)g\approx -\rho_{0}\beta \left(T-T_{0}\right)g$$

where $\rho_{0}$ is fluid density, $T_{0}$ is ambient temperature, β is thermal expansion coefficient. It can be obtained that the Grashof number $Gr=\frac{g\alpha_{v}\Delta TL^{3}}{v^{2}}$ is approximately equal to 47, so the flow is laminar [33].

For the energy conservation equation, the source term is zero everywhere except at the top surface of the substrate under the laser spot. To simplify the operation without loss of generality, it is assumed that the heat loss to the outside of the reaction chamber is negligible. Moreover, we use the Gaussian-like beam which is suitable for 2D simulation, the net heat flux under the laser beam can be expressed as

(3) $$q=\alpha \frac{2P}{\pi x_{0}^{2}}\exp\left\{-\frac{2x^{2}}{x_{0}^{2}}\right\}$$

where α is the absorptivity of the laser beam at the substrate surface,P is laser power, $x_{0}\;$is the x-coordinate of the spot center and x is *x*-axis distance to spot center. Considering the absorption depth of the laser in the simulation, the additional source term method is used to treat the laser heat flux equivalently. The heat flux is treated as an internal heat source, so the source term $S_{T}$ can be expressed as

(4) $$S_{T}=\frac{q}{l_{0}}$$

where $l_{0}\;$is laser absorption depth, the 1064 nm laser absorption depth of substrate.

### 2.2. Chemical Reaction and Crystallization

In this study, the model described a two-step process in the LIDS process, as shown in Equations (5) and (6).

(5) $$A\left(\mathrm{aq}\right)+\;B\left(\mathrm{aq}\right)\;\overset{E_{a1}}{\to }\;C\left(\mathrm{aq}\right)$$

(6) $$C\left(\mathrm{aq}\right)\;\overset{\Delta G_{U},E_{a2}}{\to }\;C\left(s\right)$$

The first step is called *Homogeneous Reaction* where $E_{a1}$ is the activation energy of the reaction, described in Equation (5). In this step, precursor species A(aq) reacts with species B(aq) and produces an intermediate species C(aq) that is considered to be a dissolved phase and can diffuse. The second step is called *Crystallization* (6), where $E_{a2}$ is the corresponding active energy and $\Delta G_{U}$ is the kinetic barriers of diffusion. In this step, species C(aq) transforms into precipitate C(s) under certain conditions.

The governing Equation (1) of A and B can be written as Equations (7) and (8), whose source term $S_{1}$ is calculated by Equation (9), where $a_{1}$ is the chemical reaction constant, R is the gas constant and T is the fluid temperature.

(7) $$\frac{\partial C_{A}}{\partial t}+div\left(VC_{A}\right)=div\left(D_{A}gradC_{A}\right)-S_{1}$$

(8) $$\frac{\partial C_{B}}{\partial t}+div\left(VC_{B}\right)=div\left(D_{B}gradC_{B}\right)-S_{1}$$

(9) $$S_{1}=a_{1}exp\left(-\frac{E_{a1}}{RT}\right)C_{A}C_{B}$$

The crystallization (6) in this study includes two stages: nucleation and crystal growth. According to the classical nucleation model based on the Gibbs theory [34], the nucleation process is the process in which C(aq) aggregates beyond the critical radius $r^{*}$, that is, the process of forming nuclei. The growth process is the process of continuing to grow on the aforementioned nuclei. In this study, we consider heterogeneous nucleation on the solid-fluid interface and assume that the solid surface nucleation process occurs after the model-defined supersaturation is reached. The governing Equations (1) of C(aq) are shown in the Equation (10):

(10) $$\frac{\partial C_{C}}{\partial t}+div\left(VC_{C}\right)=div\left(D_{C}gradC_{C}\right)-S_{2}+S_{1}$$

where $S_{2}$ is source term due to consumption during the deposition process, as shown in Equation (11)

(11) $$S_{2}=\min\left(1,\frac{N_{hnn}}{\frac{L_{0}}{r^{*}}}\right)a_{2}exp\left(-\frac{E_{a2}}{RT}\right)\;\Theta C_{C}\left(C_{C}-C_{C_{s}}\right)+\frac{dN_{hnn}}{dt}\frac{4}{3}\pi r^{*}{}^{3}\rho \frac{1}{M_{C}}$$

It represents the consumption of C (aq) per unit time, which includes two parts: the first part is the consumption caused by nucleation and it means the C (aq) consumption of crystal nuclei reaching the critical radius $r^{*}$; the second part represents the consumption caused by the growth of the crystal nucleus. min is the minimum function, $N_{hnn}$ is the number of nuclei, $L_{0}$ is the characteristic length and the term $\min\left(1,\frac{N_{hnn}}{\frac{L_{0}}{r^{*}}}\right)$ means that the number of nuclei has an effect on the growth rate. While the number of nuclei reaches a maximum in a characteristic length region, it is considered that the number of nuclei no longer affects the deposition rate. Θ is a step function. Here, when $C_{C}$ is greater than $C_{C_{S}}$ (the saturated concentration of C in the solution), it is taken as 1, and in other cases, it is taken as 0. $N_{hnn}$ is determined by Equation (12), where $\Delta G_{U}$ is Desolvation energy, and the detailed expressions are in the Supplementary Materials Section 1.1.

(12) $$N_{hnn}=\Theta \int 4\pi r^{*2}C_{C}^{2}p\exp\left(-\frac{\Delta G_{U}}{kT}\right)\Gamma \exp\left(-\frac{\Delta G^{*}}{kT}\right)dt$$

## 3. Numerical Method

**Figure 2:**
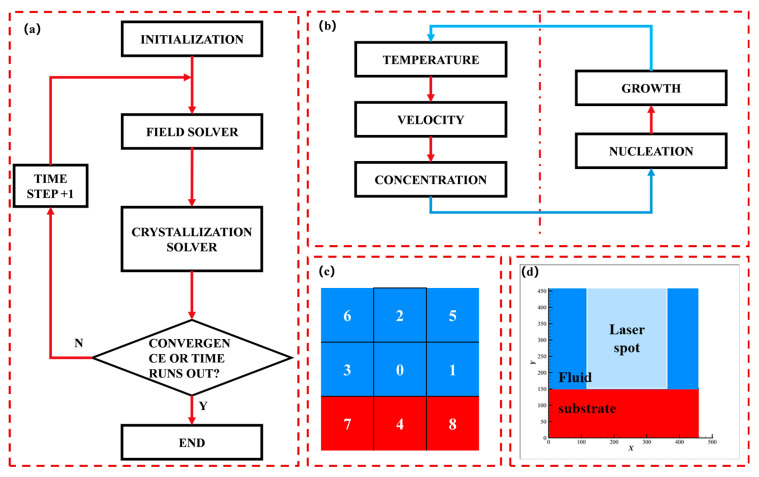
Calculation framework and model. (**a**) Flowchart of calculation program; (**b**) Internal logical relationship between field solver and crystallization solver; (**c**) Schematic diagram of the computational lattice at the fluid–solid interface; (**d**) Schematic diagram of computational domain.

The structure of the calculation program in this paper is shown in Figure 2a. The main process is the flow field solution and the crystallization solution. Its internal structure is illustrated in Figure 2b. In a calculation cycle, first, the temperature field is solved and the temperature is used as a known condition to solve the flow, then the concentration distribution is solved, and the flow and mass transfer process only takes place in the fluid lattice. Then, the solution of the nucleation and growth processes are solved. After the loop ends, it is determined whether the convergence or reach the end time of the simulation, so as to perform the next time step calculation or end the calculation.

### 3.1. Simulation of Multiple Physical and Chemical Fields by Lattice Boltzmann Method

For the multiple fields simulation, we use multi-distribution method of LBM [22], which involves the solution of flow, heat transfer and mass transfer Equations (1). First, this paper takes the solution of the flow field as an example to illustrate the solution process. Follow the lattice Bhatager–Gross–Krook (LBGK) equations in (13)

(13) $$f_{i}\left(x+c_{i}\delta t,t+\delta t\right)-f_{i}\left(x,t\right)=-\frac{\delta t}{\tau }\left[f_{i}\left(x,t\right)-f_{i}^{eq}\left(x,t\right)\right]+S_{i}\delta t$$

where $f_{i}\left(x,\;t\right)$ is the velocity distribution function in the ith direction with velocity vector $c_{i}$ at lattice site vector ***x*** and time *t*, Δt is the time increment, and $\tau =\nu /c_{s}^{2}+0.5\;$is the collision time, where ν kinematic viscosity,$\;c_{s}={\left(RT\right)}^{\frac{1}{2}}$ is speed of sound. In this study, we use D2Q9 LBGK equations, where *i* takes 0 to 8, representing the discrete velocity in nine directions, $c_{s}$ is equal to $\frac{1}{\sqrt{3}}$, $S_{i}$ denotes the source term due to the external force, for a buoyancy vector ***F*** of shape (2), the source term for the second-order accuracy can be

(14) $$S_{i}=\left(1-\frac{1}{2\tau }\right)\omega_{i}\left(\frac{c_{i}-u}{c_{s}^{2}}+\frac{\left(c_{i}\cdot u\right)c_{i}}{c_{s}^{4}}\right)\cdot F$$

where, $\omega_{i}$ is weight function in the ith direction. The equilibrium velocity distribution function $f_{i}^{eq}$ takes the following form

(15) $$f_{i}^{eq}=\omega_{i}\rho \left[1+\frac{c_{i}\cdot u}{c_{s}^{2}}+\frac{{\left(c_{i}\cdot u\right)}^{2}}{2c_{s}^{4}}-\frac{u\cdot u}{2c_{s}^{2}}\right]$$

According to the above theory, the lattice Boltzmann algorithm (named by algorithm I) for solving the flow can be obtained as follows:

1. Determine the force density ***F*** for the time step in Equation (2).
2. Compute the fluid density and velocity.
3. Compute the equilibrium populations $f_{i}^{eq}\;$in Equation (15) to construct the collision operator.
4. Compute the source term$\;S_{i}$ in Equation (14).
5. Apply collision and source to find the post-collision populations and propagate populations.

Based on the above algorithm, we can solve the flow field in LIDS, and the same algorithm can also be used to solve the temperature and concentration fields. For the solving of the concentration field, we introduce three concentration distribution functions $g_{1i}$*,* $g_{2i},$ $g_{3i}$ correspond to the three components A, B, and C respectively. It turns out that the LBGK equations of *m*th species is:

(16) $$g_{m}{}_{i}\left(x+c_{i}\Delta t,t+\Delta t\right)-g_{mi}\left(x,t\right)=-\frac{1}{\tau_{mg}}\left(g_{mi}\left(x,t\right)-g_{mi}^{eq}\left(x,t\right)\right)+Q_{mi}\left(x,t\right)$$

where the suitable source terms $Q_{mi}$ solves the ADE for the *m*th concentration $C_{m}=\sum_{i}g_{i}$. The relation between transport coefficient $D_{m}$ and relaxion time is

(17) $$\tau_{mg}=\frac{D_{m}}{c_{s}^{2}}+\frac{1}{2}$$

The equilibrium distribution typically assumes the form

(18) $$g_{mi}^{eq}=\omega_{i}C_{m}\left[1+\frac{c_{i}\cdot u}{c_{s}^{2}}+\frac{{\left(c_{i}\cdot u\right)}^{2}}{2c_{s}^{4}}-\frac{u\cdot u}{2c_{s}^{2}}\right]$$

As for species source term, using a similar approach to the NSE, we can redefine the macroscopic moments according to the reference [22].

(19) $$C_{m}=\underset{i}{\sum }g_{mi}+\frac{Q_{mi}\Delta t}{2},Q_{mi}=\left(1-\frac{1}{2\tau_{g}}\right)w_{mi}S_{C}$$

where $S_{C}$ is source term. When the species to be solved is A or B, it takes equation $-S_{1}$; when the species to be solved is *C*, it takes equation $S_{1}-S_{2}$.

The solution of the energy conservation equation is more complicated than the solution of the component transport equations because it involves conjugate heat transfer. In this study, we use the unsteady conjugate heat transfer LBM model in reference [35], which uses the a sensible-enthalpy-like quantity $h^{*}={\left(\rho c_{p}\right)}_{0}T$, where ${\left(\rho c_{p}\right)}_{0}$ is reference heat capacitance const. the distribution function $h_{i}\;$and the macroscopic temperature *T* can be written as

(20) $$T=\frac{h^{*}}{{\left(\rho c_{p}\right)}_{0}}=\frac{1}{{\left(\rho c_{p}\right)}_{0}}\;\underset{i}{\sum }h_{i}$$

The equilibrium distribution reads:

(21) $$h_{i}^{eq}=\omega_{i}h^{*}\left[1+\frac{c_{i}\cdot u}{c_{s}^{2}}+\frac{{\left(c_{i}\cdot u\right)}^{2}}{2c_{s}^{4}}-\frac{u\cdot u}{2c_{s}^{2}}\right]$$

The relation between thermal diffusivity $\frac{\lambda }{\rho c_{p}}$ and relaxion time is

(22) $$\tau_{h}=\frac{\lambda }{\rho c_{p}c_{s}^{2}}+\frac{1}{2}$$

Defining $\sigma =\frac{\rho c_{p}}{{\left(\rho c_{p}\right)}_{0}}$ as the ratio of heat capacitance, the extra source item caused by conjugate heat transfer between fluid and solid is:

(23) $$P_{i}=\frac{\omega_{i}}{c_{s}^{2}}\sigma \left(1-\frac{1}{2\tau }\right)\left(\underset{j}{\sum }\left[g_{j}\left(x_{\alpha },t\right)-g_{j}^{eq}\left(x_{\alpha },t\right)\right]c_{j\alpha }\right)\left(\underset{j}{\sum }\frac{\omega_{j}}{\sigma }\left(x_{\alpha }+c_{j\alpha }\right)c_{j\alpha }\right)\;\;-\frac{\omega_{i}}{c_{s}^{2}}\frac{h^{*}}{\sigma }u_{\alpha }\underset{j}{\sum }\omega_{j}\sigma \left(x_{\alpha }+c_{j\alpha }\right)c_{j\alpha }$$

The Einstein summation convention is used here, and the dimension index α can be 1 or 2. One can observe that in the present work the calculation of $P_{i}$ is a completely local operation. Away from the fluid–solid interface, the output of Equation (23) becomes zero since there is no spatial variation of the heat capacitance in a homogenous material layer. Finally, we can get the source term of (16) for energy conservation equation:

(24) $$Q_{i}=P_{i}+\left(1-\frac{1}{2\tau_{g}}\right)w_{i}S_{T}$$

At this point, we can use a similar algorithm Ⅰ to solve the multi-field coupling problem in LIDS. As shown in the left half of Figure 2b, the temperature, velocity, and concentration fields are solved in order. At last, based on the simulation conditions in this paper, and the absorptivity of the 1064 nm wavelength laser, we can assume that the laser absorption depth is 50 lattice depths under the simulation conditions, that is, the heat source is applied in the range of 50 lattice depths within the radius of the spot. As the deposition process progresses, the boundaries of the substrate move, and thus the location of the heat source moves, so at the end of each computation cycle, the heat source is reassigned at lattices to match the physical reality. This treatment method can consider the upward movement of the laser absorption surface due to material deposition to reduce the error of the simulation process.

### 3.2. The Computation of Crystallization Process

As shown in Figure 2b, in a calculation cycle, after solving three fields, the Crystallization process including nucleation and growth is solved. Based on the model of literature [23,36], we propose our crystallization model suitable for the LIDS process. In the schematic diagram of the calculation lattice shown in Figure 2c, blue represents fluid lattices, red represents solid lattices, and the lattice 0 is taken as an example to explain the crystallization process at the solid-liquid interface. The nucleation process is controlled by the nucleation rate shown in Equation (11), which represents the number of reaching the critical radius in the unit volume over time. Lattice 0 is in the fluid region and contains three substances A(aq), B(aq), and C(aq). When the supersaturation of C(aq) is greater than zero, the current accumulated number exceeds the critical radius $r^{*}$. When the number of crystal nuclei is greater than or equal to one, the growth process occurs whose consumption can be calculated by Equation (11).

(25) $$m\left(t+\Delta t\right)=m\left(t\right)+M_{C}S_{2}\Delta V\Delta t$$

Equation (25) is used to simulate the sediment mass accumulated in the growth process, where ΔV is lattice volume, $M_{C}$ is molar mass. Assuming the depth of one node in the z direction (perpendicular to the paper) equals the lattice length Δ*x =* 1. When the mass of the sediment is greater than or equal to mass of substance C(s) occupying the whole lattice volume, it is transferred to a solid identification, and its parameters are changed to solid parameters (such as thermal diffusivity).

### 3.3. Computational Domain and Initial and Boundary Conditions

The calculation area is a square area of 400×400 μm, as shown in Figure 2d, which is discretized into a 460 × 460 lattices system, the thickness of the substrate area is 130 μm, and the initial concentrations of A(aq) and B(aq) are $C_{0}$, The square is initially free of intermediate species C(aq).

This simulation can be thought of as a very small area taken from a reactor much larger than the size of the simulated area. For the temperature field, constant temperature boundary conditions (T_0 = 288.15 K) are used on the left and right boundaries, and adiabatic boundary conditions are used for the rest. For flow field, no-slip wall boundary conditions are used at the fluid–solid interface, and symmetric boundary conditions are used for the rest. For concentration fields, constant flux boundary conditions are used on the top, left, and right boundaries. Since the material dissolution process is not considered, At the fluid−solid interface, the no-flux boundary condition is used.

The key parameters used for the base case are listed in Table 2, some of which are taken directly from refs [34]. The typical time required for a simulation case is about 9 to 12 h on a Dell Precision 7920 Workstation with 20 processors (Intel Xeon Platinum 8180 M) and 128 GB of memory. We selected different parameters to simulate 38 working conditions in order to obtain the key factors affecting the macroscopic morphology during the LIDS deposition. All working conditions and their parameters are placed in the Table S2. We use its serial number to represent the corresponding condition in the subsequent description. For convenience, the horizontal and vertical axes of the figures about calculation areas in this paper use dimensionless lengths, the density unit is $g/{\mathrm{cm}}^{3}$, and the units of all other parameters use the International System of Units.

## 4. Results and Discussions

### 4.1. The Coupling of Multiple Physical Fields

**Figure 3:**
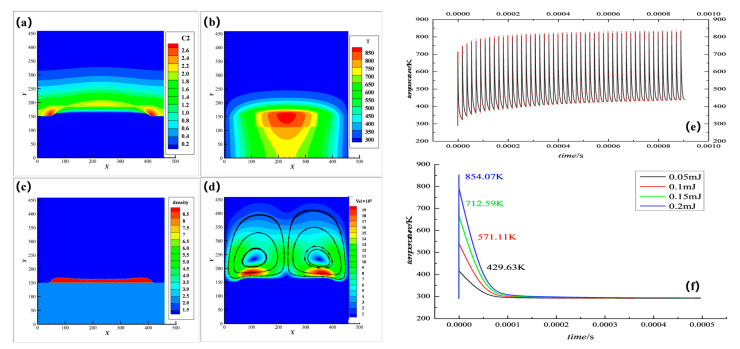
Laser-induced coupling of multiple physical fields. (**a**) C(aq) concentration distribution; (**b**) Temperature distribution cloud map; (**c**) Sediment topography; (**d**) Flow field and streamline diagram; (**e**) Temperature change curve with time; (**f**) Temperature change curve under single pulse of different laser conditions.

Figure 3 shows a typical simulation result of multiple physical fields for the LIDS process, in which (a), (b), (c), and (d) show the distribution of concentration, temperature, precipitates, and velocity, in multiple physical fields at time steps of 500,000 under a certain standard condition (labeled as condition 2 as shown in the Table S3.

The laser-induced deposition system is a complex multi physicochemical coupling process. Due to the local temperature field change induced by the pulse laser, the chemical reaction can be activated, further making the deposition occur. Meanwhile, with the accumulation of heat, the local high temperature leads to the natural convection as shown in Figure 3d, and the maximum velocity is around 100 μm/s. One role of the natural convection is to accelerate the material transport from the edge to the center of the spot. Subsequently, due to the continuous formation of sediments causing the change of the flow boundary, the laser absorption region also moved upward. That is to say, the material transport and crystallization process finally in turn affect the temperature distribution.

Figure 3e shows the temperature change curve of the spot center during the first 100 pulse periods and Figure 3f shows the temperature change curve of a single pulse with different energy. It can be seen that the temperature change has a drastic change in each cycle, and tends to have the same pattern after a few cycles. Therefore, the reaction in LIDS is induced by a short laser pulsed-heating and the heat accumulation also deposition conditions.

### 4.2. A Morphology Analysis in a Typical Deposition Process

**Figure 4:**
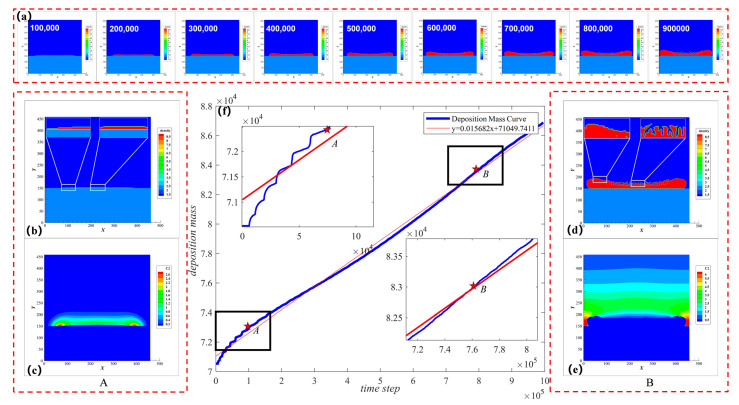
Sediment mass change curve and morphology structure change diagram during LIDS: (**a**) Change curve of dimensionless deposition mass with time; (**b**) Sediment morphology and local enlarged view corresponding to point A of the curve (**a**); (**c**) C (aq) concentration distribution corresponding to point A; (**d**) Sediment morphology and local enlarged view corresponding to point B of the curve (**a**); (**e**) C(aq) concentration distribution corresponding to point B; (**f**) The sediment morphology changes with time. From left to right, each cloud image has an interval of 100,000 time steps.

This section takes the standard condition (condition 2 in SI) as an example to illustrate the LIDS process. The deposition morphology change with time is simulated in Figure 4a. Figure 4f shows the corresponding dimensionless sediment mass change with time. The blue line in the figure is the simulated sediment variation curve, and the red line is the corresponding linear regression fitting curve. It can be seen that the coefficient of determination $R^{2}=0.9872$ is close to 1, so the slope of the fitted straight line can be used to represent the average deposition rate $R_{G}$, during the entire deposition. For all of the simulation conditions, the rate value is listed in Table S3. It can also be seen from Figure 4f that the simulation results tend to stabilize after a period of fluctuation, which indicates that the deposition rate tends to be stable. The curves in the left and right black boxes of Figure 4f and their enlarged images represent the fluctuation stage and stable stage. Here, two points A and B are selected as representatives to analyze their morphology and C(aq) concentration changes at two stages. The simulation results correspond to Figure 4b,c and Figure 4d,e, respectively.

Concentration distribution in the two stages is different, leading to different morphology of deposited films. In the first stage, as indicated by point A, the deposition rate fluctuated with pulsed heating. According to Figure 3, the chemical reaction is largely affected by the temperature change. Therefore, the former stage is a chemical reaction dominating stage. As shown in Figure 4b,c, the morphology of the sediments at this stage is a unimodal structure as a whole, and its local roughness is small, the deposition rate is relatively slow, and the concentration supply is relatively sufficient at this time. After more than ten pulse cycles, the fluctuation gradually decays, indicating a deposition process with a constant reaction rate. In the second stage indicated by point *B*, the deposition rate tends to stabilize, as shown in Figure 4d,e, the overall morphology of the sediment changed to a bimodal structure with higher two sides and the local roughness was very high, and the deposition rate tended to be stable. At this time, the concentration near the growth interface was very low.

### 4.3. Factors Affecting Sediment Morphology

#### 4.3.1. Evolution of Overall Morphology and Structure

**Figure 5:**
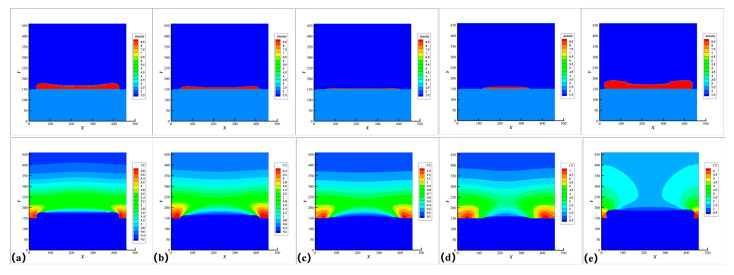
Five typical sediment morphologies and their concentration cloud diagram of C(aq) when the time step is 500,000 steps. upper: morphology, lower: concentration. (**a**) standard condition; (**b**) moderate growth condition; (**c**) low deposition rate condition; (**d**) the condition affected mainly by temperature; (**e**) condition with small roughness.

In order to further understand the relationship among process-condition-materials, we use the sediment roughness ($R_{a})$ and the overall trend (ε) to quantitively analyze the morphology of the precipitates. The overall trend (ε) represents the overall trend of the sediment morphology. The larger the absolute value, the greater the fluctuation and when it is negative, it means the sediment has a unimodal structure; on the contrary, when it is positive, it means the sediment has a bimodal structure. The more specific definition and calculation method are in the Supplementary Materials Section 1.2. Five typical sediment morphologies are summarized, as shown in Figure 5a representing a faster deposition rate and larger local roughness as the standard condition; Figure 5b representing a moderate growth rate with a smooth surface roughness; Figure 5c representing a process with a very low average deposition rate; Figure 5d represents a process that is more affected by temperature, whose deposits are concentrated in the area close to the spot, the roughness $R_{a}$, overall trend ε and average deposition rate $R_{G}$ of e are much larger than that of d; Figure 5a,e are similar but their local roughness is smaller. Their *ε* and $R_{a}$ of each five condition is calculated in Table 3.

As shown in Figure 5 and Table 3, the laser-induced chemical deposition system is a complex nonlinear system, which involves a large number of parameters, including the parameters of the system itself, environmental parameters, and so on. In the following sections, we will discuss the effects of these parameters individually.

**Figure 6:**
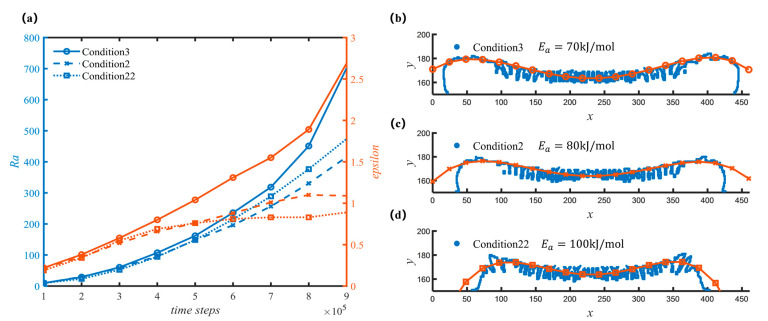
Morphology of sediments with different homogeneous reaction activation energy. (**a**) Curves of roughness and overall trend with different working conditions; (**b**) Sediment profile and fit line for condition 3 at 500,000 time steps; (**c**) Sediment profile and fit line for condition 2 at 500,000 time steps; (**d**) Sediment profile and fit line for condition 22 at 500,000 time steps.

Firstly, as shown in Figure 6, the changes in sediment morphology are obtained by adjusting a single parameter under the condition of keeping the other parameters unchanged, respectively, and the curve in Figure 6a shows the change in sediment roughness, $R_{a}$, and the overall trend, ε, the Figure 6b–d on the right show the corresponding sediment profile and fitting curve.

Figure 6 shows the change in sediment morphology with the $E_{a1}$. At the same temperature, higher $Ea_{1}$ indicated a slower reaction. It can be found that due to the difference in $E_{a}$, the overall morphology of the sediment is greatly affected. The corresponding average deposition rates are 0.01565, 0.01952, and 0.001103 (see Table S3), and the deposition rate gradually decreases, which is consistent with the physical model. At the same time, due to the reduction of $E_{a}$, the overall morphology of the sediment has also changed, that is, the sediment is more concentrated in the center of the spot. $E_{a}$ mainly affects the inhomogeneity of the sediment, the smaller the $E_{a}$, the greater the inhomogeneity, and the local roughness also increases slightly.

**Figure 7:**
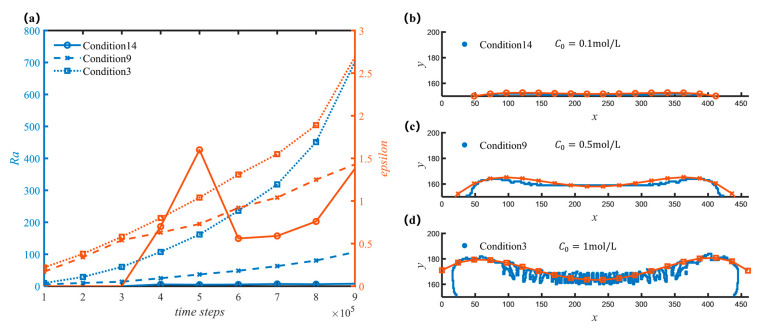
Morphology of sediments with different initial concentration. (**a**) Curves of roughness and overall trend with different working conditions; (**b**) Sediment profile and fit line for condition 14 at 500,000 time steps; (**c**) Sediment profile and fit line for condition 9 at 500,000 time steps; (**d**) Sediment profile and fit line for condition 3 at 500,000 time steps.

**Figure 8:**
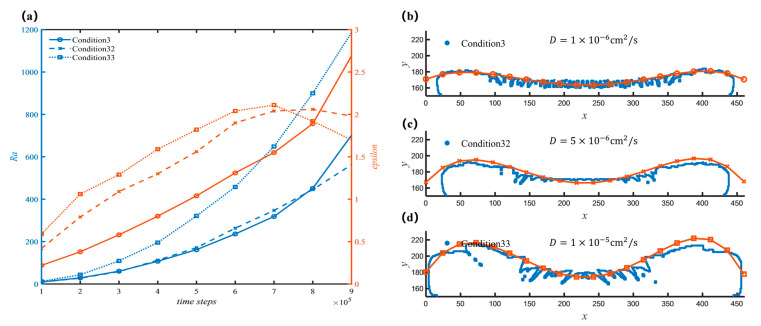
Morphology of sediments with different diffusion coefficients. (**a**) Curves of roughness and overall trend with different working conditions; (**b**) Sediment profile and fit line for condition 3 at 500,000 time steps; (**c**) Sediment profile and fit line for condition 32 at 500,000 time steps; (**d**) Sediment profile and fit line for condition 33 at 500,000 time steps.

Figure 7 shows that the initial concentration $C_{0}$ of A and B has a great influence on the overall morphology of the sediment. Figure 8 shows the morphology and structure of the deposits formed when C0 is 0.1, 0.5, and 1 mol/L, respectively, and the average dimensionless average deposition rates are 0.00108, 0.00777, and 0.01952, respectively. As $C_{0}$ increases, the average deposition rate also increases sharply, thus, the larger the $C_{0}$, the greater the deposition quality at the same time; as shown in Figure 8a. In addition, with the increase of $C_{0}$, the roughness increases sharply. Comparing the morphology of different $E_{a1}$ shown in Figure 6, for the influence of the overall morphology, $C_{0}$ mainly affects the distribution in the x-axis. It can be seen from the graph in Figure 8a that the epsilon curve jumps when $C_{0}=0.1\;\mathrm{mol}/L$. In contrast with Figure 8b, due to the small deposition mass, the very small increase in the deposition mass will greatly change the overall topography and thus the resulting epsilon value will be greatly affected.

Morphology is also affected by the mass transfer process. Here we use different diffusion coefficients to investigate the effect of the diffusion condition. Figure 8 shows the deposition morphologies when the diffusion coefficients are $1\times 10^{-6}$, $5\times 10^{-6}$, and $1\times 10^{-5}$. The average deposition rates were 0.01952, 0.02695, and 0.03068, respectively, which increased with the increase of the diffusion coefficient. Combined with the results shown in Figure 8, it can be found that the larger the diffusion coefficient of the material, the corresponding local roughness will not necessarily keep increasing. For the case of a small diffusion coefficient, more small structures will appear on the surface of the deposited material, which makes the roughness increase, as the diffusion coefficient increases, the number of fine structures decreases, the curve becomes smooth and the roughness decreases. However, further increasing the diffusion coefficient, as shown in Figure 8d, larger structures appear, making the overall roughness instead increase. Although in reality, the diffusion coefficient will not change a lot for a certain condition, it can be concluded that the mass transfer is an important factor affecting the size of the local structure. When other parameters of the reaction system are suitable, promoting the mass supply can obtain finer forest-like sediments.

**Figure 9:**
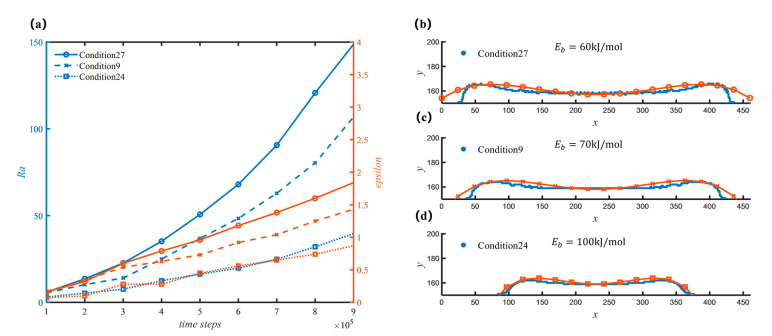
Morphology of sediments with different Growth barrier. (**a**) Curves of roughness and overall trend with different working conditions; (**b**) Sediment profile and fit line for condition 27 at 500,000 time steps; (**c**) Sediment profile and fit line for condition 9 at 500,000 time steps; (**d**) Sediment profile and fit line for condition 24 at 500,000 time steps.

From the consumption side, Figure 9 shows that the consumption of C(aq) also has a great influence on the sediment morphology. At a certain temperature, lower Ea2 means a lower barrier for precursor consumption. Compared with the effect of $E_{a}$ on the deposition rate and sediment morphology, the effect of $E_{a2}$ is similar to that of $E_{a1}$. That is, with increasing $E_{a2}$, the average deposition rate gradually decreases (0.00825, 0.00777, 0.00548, respectively). Comparing to Figure 7, it can be seen that the distribution along the surface of the substrate is more concentrated in the center of the light spot with the increase of $E_{a2}$.

#### 4.3.2. Local Condition for Microstructure

**Figure 10:**
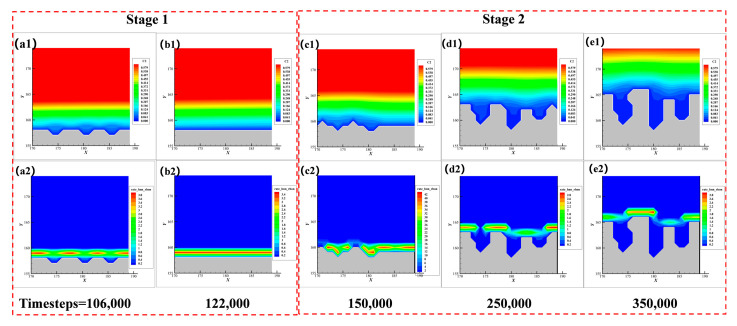
Change of local roughness with time: (**a1**–**e1**) Concentration distribution of reactants for the corresponding time step; (**a2**–**e2**) Precursor consumption rate corresponding to time step.

Figure 10 is a comparison of the deposition morphology of the local structure at different times based on the standard condition. The upper figures show the concentration distribution of A(aq), the lower figures represent the relative consumption rate of C (aq) at the current moment, and the gray part in the figure represents the precipitation.

We can see a two-stage evolution of the local structure. In the first stage, as shown in Figure 10a,b, the local roughness is small, and the depressions of the microstructure can grow and become smooth. Comparing Figure 10(a1) to Figure 10(b1), the concentration of reactants near the solid-liquid interface is higher. Comparing Figure 10(a2) to Figure 10(b2), the deposition rate in the depressions is comparable to that in the peaks. Therefore, the deposition is in the reaction-limiting stage and the deposition rate is uniform.

In the second stage, as shown in Figure 10c1–e1, with the progress of the deposition process, the concentration of reactants near the sediment has an obvious boundary layer, resulting in different position competition for C(aq), the material supply is insufficient, and the deposition becomes supply-limiting. As shown in Figure 10c2–e2 in the depression of the microstructures, which are far from the mainstream supply area, the deposition rate is slowed down. Over time, the growth of local fine peaks in turn further inhibits the supply of reactants in the depression, so the local roughness also becomes larger.

In conclusion, the local roughness is largely related to the competition between material supply and precursor consumption. When the deposition is controlled by the reaction, the sediment tends to grow isotropically, and the roughness is relatively small. When the material supply becomes the rate-limiting factor, the anisotropy in the deposition process is more prominent and the sediment is rougher. This also provides a reference for controlling the sediment morphology in the experiment.

#### 4.3.3. Sediment Topography Control Method

According to the results discussed above, a guidance for the morphology control of the deposited materials in the LIDS process was summarized in Table 4. The sediment morphology can be controlled by tuning key parameters. Process parameters include energy barrier $E_{a}$, initial concentration$\;C_{0},\;$and diffusion coefficient D, the topographic feature value include average deposition rate $R_{G}$,roughness $R_{a}$, overall trend ε and Width in substrate. The diffusion coefficient D represents the process of diffusion and material supply, and the $\frac{E_{a}}{kT}$ value (including $\frac{E_{a1}}{kT}$,$\;\frac{E_{a2}}{kT}$) often represents the reaction rate or deposition rate. In the actual reaction process, after a given reaction system, D and the $E_{a}$ of each process are difficult to change, so we can tune the deposition morphology of materials by adjusting environmental conditions such as temperature and initial concentration. In the analysis described below, an increase in $E_{a}$ can be equivalent to a decrease in ambient temperature or a decrease in reaction rate.

**Figure 11:**
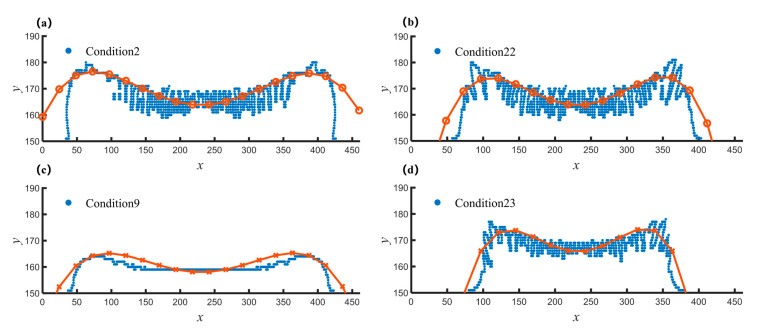
Examples of parameters control for desired morphology. (**a**) Sediment profile and fit line for condition 2 at 500,000 time steps; (**b**) Sediment profile and fit line for condition 22 at 500,000 time steps; (**c**) Sediment profile and fit line for condition 9 at 500,000 time steps; (**d**) Sediment profile and fit line for condition 23 at 500,000 time steps.

Here, we illustrate two examples to show how to tune the morphologies of deposited materials according to Table 4. The sediment morphology under standard conditions is used as a reference, as shown in Figure 11a. If we want to reduce the surface roughness $R_{a}$ and increase the overall trend ε of sediment, according to Table 4, we can choose to reduce the initial concentration $C_{0}$ and appropriately increase the reaction activation energy $E_{a1}$. The deposition morphology is shown in Figure 11c corresponding to condition 9. Combining with Table 3, we can see that the surface roughness $R_{a}$ decreases, and the ε value increases. Another case is that Figure 11c shows the sediment morphology of working condition 9. If we need to increase the surface roughness $R_{a}$ while keeping the overall trend ε unchanged, according to Table 4, we can choose to appropriately increase the initial concentration $C_{0}$ and the reaction activation energy $E_{a1}$, corresponding to the working condition 23 shown in Figure 11b, which can meet our needs. Further, if we want to increase the roughness and reduce ε based on condition 9, we can continue to increase $E_{a1}$, as shown in Figure 11d, combined with Table 3, we can see that our requirements for morphology are satisfied.

## 5. Conclusions

A simulation model based on a LBM is established to study the LIDS process. The LIDS involves multi-filed interactions of process parameters, conditions, and materials. The simulation results indicated that the most influential factors in different stages of crystallization vary. Generally, the first stage is dominated by the chemical reaction, and the second stage is rate-limited by the materials transport. By analyzing typical sediment morphologies, we discussed their most affecting factors, which are the activation energy for growth, diffusion coefficient, and initial concentration. Then we analyzed the essential cause of local roughness change, which is due to the local competition for the supply of the precursor. A method for sediment topography control was developed, facilitating LIDS as a precise synthesis technology.

## Figures and Tables

**Table 1:** Values of different governing equations.

Equation	ρ	ϕ	$\Gamma_{\phi }$	$S_{\phi }$
Continuity Equation	ρ	1	0	0
Momentum Equation (x direction)	ρ	u	μ	$\rho f_{x}-\frac{\partial p}{\partial x}$
Momentum Equation (y direction)	ρ	v	μ	$\rho f_{y}-\frac{\partial p}{\partial y}$
Energy Equation	$\rho c_{p}$	T	λ	$S_{T}$
Species Equation	1	C	D	S

**Table 2:** Physical and Chemical Parameters.

Parameter Name	Symbol	Unit	Value	Parameter Name	Symbol	Unit	Value
*Scale Factor of time*	$C_{t}$	s	1.99 × 10^−8^	*Molar mass of silicon*	$m_{1}$	$\mathrm{kg}\cdot {\mathrm{mol}}^{-1}$	0.028
*Scale Factor of length*	$C_{L}$	m	8.70 × 10^−7^	*Density of copper*	$\rho_{2}$	$\mathrm{kg}\cdot m^{-3}$	8960
*Scale Factor of density*	$C_{\rho }$	$\mathrm{kg}\cdot m^{-3}$	199.82	*Thermal conductivity of copper*	$k_{2}$	$W\cdot m^{-1}\cdot K^{-1}$	401
*Scale Factor of temperature*	$C_{T}$	K	28.815	*Specific heat capacity of copper*	$c_{2}$	$J\cdot {\mathrm{kg}}^{-1}\cdot K^{-1}$	390
*Scale Factor of amount of substance*	$C_{mol}$	mol	1.00 × 10^−6^	*Molar mass of copper*	$m_{2}$	$\mathrm{kg}\cdot {\mathrm{mol}}^{-1}$	0.064
*Simulation area length*	*L*	m	0.0004	*Diffusion coefficient*	*D*	$m^{2}\cdot s^{-1}$	1.00 × 10^−6^
*Gas constant*	R	$J\cdot {\mathrm{mol}}^{-1}\cdot K^{-1}$	8.314	*Homogeneous Reaction Activation Energy*	$E_{a1}$	$J\cdot {\mathrm{mol}}^{-1}$	80,000
*laser pulse repetition rate*	Rep	Hz	50,000	*Chemical reaction rate constant*	$a_{1}$		3.80 × 10^9^
*Laser pulse width*	*w*	s	1.00 × 10^−7^	*The saturation concentration of copper in water*	$C_{s2}$	$\mathrm{mol}\cdot m^{-3}$	8.00 × 10^−16^
*Laser peak power*	*P*	$W\cdot m^{-3}$	1.11 × 10^−9^	*Growth barrier*	$E_{a2}$	$J\cdot {\mathrm{mol}}^{-1}$	70,000
*Density of water*	$\rho_{0}$	$\mathrm{kg}\cdot m^{-3}$	999.1	*growth constant*	$a_{2}$		1.00 × 10^9^
*Thermal conductivity of water*	$k_{0}$	$\mathrm{kg}\cdot m^{-3}$	0.62	*Contact angle*	θ	rad	0.15
*Thermal expansion coefficient of water*	$\beta_{0}$	—	0.00289	*diffusion barrier*	$E_{u}$	$J\cdot {\mathrm{mol}}^{-1}$	80,000
*Specific heat capacity of water*	$c_{0}$	$J\cdot {\mathrm{kg}}^{-1}\cdot K^{-1}$	4189	*Molecular Collision Frequency*	ω	Hz	2.00 × 10^13^
*Molar mass of water*	$m_{0}$	$\mathrm{kg}\cdot {\mathrm{mol}}^{-1}$	0.018	*Molecular mean free path*	$l_{0}$	m	3.00 × 10^−10^
*Dynamic viscosity of water*	μ	$N\cdot \;s\cdot m^{-2}$	0.00113757	*molecular volume*	$v_{0}$	$m^{3}$	1.41 × 10^−29^
*Density of silicon*	$\rho_{1}$	$\mathrm{kg}\cdot m^{-3}$	2330	*Copper-Silicon-Water Surface Energy*	σ	$J\cdot m^{-2}$	0.1
*Thermal conductivity of silicon*	$k_{1}$	$W\cdot m^{-1}\cdot K^{-1}$	149	*Laser penetration depth*	$l_{p}$	—	50
*Specific heat capacity of silicon*	$c_{1}$	$J\cdot {\mathrm{kg}}^{-1}\cdot K^{-1}$	706.75	*Initial concentration of reactants A or B*	$C_{0}$	$J\cdot {\mathrm{mol}}^{-1}$	10,000

**Table 3:** Sediment morphologies under different conditions when the time step is 500,000 steps.

Label	Condition Number in SI	$R_{a}$	ε	$R_{G}$
a	2	147.82	0.76	0.015654
b	9	36.83	0.73	0.019684
c	16	9.96	0.83	0.002442
d	25	10.10	0.06	0.004158
e	32	171.14	1.56	0.269507

**Table 4:** Sediment topography control method.

Process Parameter	Topographic Feature Value			
	Average Deposition Rate	Roughness	Overall Trend	Width in Substrate
$E_{a}$	--	--	--	--
$C_{0}$	++	+++	+	+
D	++	+	+	+

## Data Availability

The authors confirm that the data supporting the findings of this study are available within the article and its supplementary materials.

## Supplementary Materials

The following supporting information can be downloaded at: https://www.mdpi.com/article/10.3390/nano12183213/s1, Figure S1: the outline of precipitation and its fit curve; Figure S2: the concentration distribution of A(aq) under different conditions; Figure S3: the concentration distribution of C(aq) under different conditions; Figure S4: the diagram of Morphology under different conditions; Table S1: Physical and Chemical Parameters; Table S2: The condition name and its detailed parameters; Table S3 Sediment morphology parameter values under different conditions.

## References

[1] Yang G., Park S.-J. (2019). Conventional and microwave hydrothermal synthesis and application of functional materials: A review. Materials.

[2] Ansari M.Z., Seo K.-M., Kim S.-H., Ansari S.A. (2022). Critical Aspects of Various Techniques for Synthesizing Metal Oxides and Fabricating Their Composite-Based Supercapacitor Electrodes: A Review. Nanomaterials.

[3] Dippong T., Levei E.A., Cadar O. (2021). Recent Advances in Synthesis and Applications of MFe_2_O_4_ (M = Co, Cu, Mn, Ni, Zn) Nanoparticles. Nanomaterials.

[4] VKochemirovsky A., Menchikov L.G., Safonov S.V., Bal’makov M.D., Tumkin I.Y.I., Tver’yanovich Y.S. (2011). Laser-induced chemical liquid phase deposition of metals: Chemical reactions in solution and activation of dielectric surfaces. Russ. Chem. Rev..

[5] Sugioka K. (2020). Handbook of Laser Micro-and Nano-Engineering.

[6] Hong S., Lee H., Yeo J., Ko S.H. (2016). Digital selective laser methods for nanomaterials: From synthesis to processing. Nano Today.

[7] Yeo J., Hong S., Kim G., Lee H., Suh Y.D., Park I., Grigoropoulos C.P., Ko S.H. (2015). Laser-Induced Hydrothermal Growth of Heterogeneous Metal-Oxide Nanowire on Flexible Substrate by Laser Absorption Layer Design. ACS Nano.

[8] Liu Z., Liu C.R. (2013). Laser induced chemical solution deposition of nanomaterials: A novel process demonstrated by manufacturing SnO_2_ nanotubes. Manuf. Lett..

[9] Manshina A., Povolotskiy A., Ivanova T., Kurochkin A., Tver’yanovich Y., Kim D., Kim M., Kwon S. (2006). CuCl2-based liquid electrolyte precursor for laser-induced metal deposition. Laser Physics Lett..

[10] Morita T. (2010). Piezoelectric materials synthesized by the hydrothermal method and their applications. Materials.

[11] Ding M., Guo Z., Zhou L., Fang X., Zhang L., Zeng L., Xie L., Zhao H. (2018). One-dimensional zinc oxide nanomaterials for application in high-performance advanced optoelectronic devices. Crystals.

[12] Abbas M., Zeng L., Guo F., Rauf M., Yuan X.-C., Cai B. (2020). A Critical Review on Crystal Growth Techniques for Scalable Deposition of Photovoltaic Perovskite Thin Films. Materials.

[13] Liu S., Liu C.R. (2019). Morphology control by pulsed laser in chemical deposition illustrated in ZnO crystal growth. Cryst. Growth Des..

[14] Liu Z., Liu C.R. (2018). Nucleation of hematite nanocrystals revealed by a single nanosecond laser pulse method. Nanoscale.

[15] Liu S., Ou C.Y., Liu C.R. (2020). Temporal and spatial temperature modelling for understanding pulsed laser induced solution based nanomanufacturing. Nanotechnology.

[16] Wang B., Jiang Y., Xu C. (2020). Phase Transition in Iron Thin Films Containing Coherent Twin Boundaries: A Molecular Dynamics Approach. Materials.

[17] Isaev V.A., Grishenkova O.V., Kosov A.V., Semerikova O.L., Zaikov Y. (2021). Simulation of 3D Electrochemical Phase Formation: Mixed Growth Control. Materials.

[18] Caiazzo F., Alfieri V. (2019). Simulation of Laser-assisted Directed Energy Deposition of Aluminum Powder: Prediction of Geometry and Temperature Evolution. Materials.

[19] Otto A., Koch H., Vazquez R.G. (2012). Multiphysical simulation of laser material processing. Phys. Procedia.

[20] Hafiz O.M., Singh A. (2011). CFD simulation of laser enhanced modified chemical vapor deposition process. Chem. Eng. Res. Des..

[21] Guo L., Geng S., Gao X., Wang W. (2021). Numerical simulation of heat transfer and fluid flow during nanosecond pulsed laser processing of Fe78Si9B13 amorphous alloys. Int. J. Heat Mass Transf..

[22] Krüger T., Kusumaatmaja H., Kuzmin A., Shardt O., Silva G., Viggen E.M. (2017). The Lattice Boltzmann Method. Principles and Practice.

[23] Chen L., Kang Q., He Y.L., Tao W.Q. (2012). Mesoscopic study of the effects of gel concentration and materials on the formation of precipitation patterns. Langmuir.

[24] Chen L., Kang Q., He Y.-L., Tao W.-Q. (2012). Pore-scale simulation of coupled multiple physicochemical thermal processes in micro reactor for hydrogen production using lattice Boltzmann method. Int. J. Hydrogen Energy.

[25] Mao Y., Xu M. (2015). Lattice Boltzmann numerical analysis of heat transfer in nano-scale silicon films induced by ultra-fast laser heating. Int. J. Therm. Sci..

[26] Cattenone A., Morganti S., Auricchio F. (2020). Basis of the lattice Boltzmann method for additive manufacturing. Arch. Comput. Methods Eng..

[27] Huang Z., Huang Y., Chen Y., Deng Y., Zhao Z. (2018). Direct and opposite droplet ejections from metal films induced by nanosecond laser pulses: Experimental observation and lattice Boltzmann modeling. J. Micromech. Microeng..

[28] Tumkin I.I., Kochemirovsky V.A., Bal’makov M.D., Safonov S.V., Zhigley E.S., Logunov L.S., Shishkova E.V. (2015). Laser-induced deposition of nanostructured copper microwires on surfaces of composite materials. Surf. Coat. Technol..

[29] Zhao Z., Yang G., Zhao K. (2022). 3D Printing of Mg-Based Bulk Metallic Glasses with Proper Laser Power and Scanning Speed. Metals.

[30] Guessasma S., Belhabib S., Benmahiddine F., Hamami A.E.A., Durand S. (2022). Synthesis of a Starchy Photosensitive Material for Additive Manufacturing of Composites Using Digital Light Processing. Molecules.

[31] Karniadakis G., Beskok A., Aluru N. (2006). Microflows and Nanoflows: Fundamentals and Simulation.

[32] Chen G. (2005). Nanoscale Energy Transport and Conversion: A Parallel Treatment of Electrons, Molecules, Phonons, and Photons.

[33] Bergman T.L., Bergman T.L., Incropera F.P., Dewitt D.P., Lavine A.S. (2011). Fundamentals of Heat and Mass Transfer.

[34] Markov I.V. (2016). Crystal Growth for Beginners: Fundamentals of Nucleation, Crystal Growth and Epitaxy.

[35] Chen S., Yan Y., Gong W. (2017). A simple lattice Boltzmann model for conjugate heat transfer research. Int. J. Heat Mass Transf..

[36] Büki A., Kárpáti-Smidróczki É., Zrínyi M. (1995). Two dimensional chemical pattern formation in gels. Experiments and computer simulation. Phys. A Stat. Mech. Its Appl..

