# Supplementary material for: Simulation of the Nucleation and Crystal Growth Process in the Laser-Induced Deposition in Solution by a Lattice Boltzmann Method
Source: Nanomaterials (Basel). 2022 Sep 16;12(18):3213. doi: 10.3390/nano12183213 (PMC9505835; doi:10.3390/nano12183213)
Supplement: Supplementary file 1 [file nanomaterials-12-03213-s001.zip › nanomaterials-1907821-supplementary.pdf]

nanomaterials

Supplementary Materials

Simulation of the Nucleation and Crystal Growth Process in the Laser-Induced Deposition in Solution by a Lattice Boltzmann Method

Yongsen He and Siyu Liu *

School of Mechanical Engineering, Shanghai Jiao Tong University, Shanghai 200240, China
* Correspondence: liusiyu@sjtu.edu.cn

Abstract: A Lattice Boltzmann model is proposed, combining the theories of nucleation and crystal growth for the study of the laser-induced deposition in solution (LIDS). The conjugate heat transfer and the natural convection of the liquid precursor were simulated with the evolving interface of crystal growth. In turn, the morphology of the deposited materials was affected by multiple process parameters, including conditions of chemical precursor and the laser-induced heat and mass transfer. Simulation results indicated that the morphology of deposited materials was mostly affected by the initial concentration of the precursor solution. Specifically, the nonuniformity of thin films was caused by the convection induced by the pulsed-laser, and the surface roughness was due to the competition of local structures for the precursor supply. A relationship of process-condition-material was established, providing guidance of choosing various parameters in LIDS for a desirable morphology of deposited material, facilitating the capabilities of pulsed lasers in precise control in nanomanufacturing.

TABLE OF CONTENTS

1 Nucleation Theory and Data Processing Methods 2
1.1 Nucleation theory 2
1.2 Definition and computational methods of Roughness and Overall trend 2
2 Detailed parameter table 3
2.1 Physical and Chemical Parameters 3
2.2 Working condition parameters 4
3 Simulation results under different working conditions 5
3.1.1 Roughness, average deposition rate and fluctuation of overall structure of sediment 5
3.1.2 Cloud map under different conditions 8

1. Nucleation Theory and Data Processing Methods

1.1. Nucleation theory

The nucleation rate formula (1) in this paper is derived from ref, which represents the nucleation rate of solute C in solution

$$J_0 = 4\pi r^{*2} C_C^2 \upsilon\lambda \exp\left(-\frac{\Delta G_U}{kT}\right) \Gamma \exp\left(-\frac{\Delta G^*}{kT}\right) \quad (1)$$

Where r^* is calculated by equation(2) and is the critical radius of the crystal at the current supersaturation,C_C is concentration of C(aq), υ is molecular collision frequency, λ is mean free path, ΔG_U is the desolvation energy, Γ is Zeldovich factor calculated by equation (5), k is the gas constant,T is temperature, ΔG^* is the nucleation energy calculated by equation (4).(5)

$$r^* = \frac{2\sigma v_1}{\Delta\mu} \quad (2)$$

Where σ is surface energy,v_1 is molecular volume, $\Delta\mu$ is supersaturation, defined by equation:

$$\Delta\mu = kTln\frac{C_C}{C_{C_s}} \quad (3)$$

Where C_{C_s} is the saturation concentration. The nucleation energy ΔG^* is:

$$\Delta G^* = \frac{16\pi}{3}\frac{\sigma^3 v_1^2}{\Delta\mu^2} \quad (4)$$

The Zeldovich factor is:

$$\Gamma = \frac{\Delta\mu^2}{8\pi v_1[\sigma^3\phi(\theta)kT]^{\frac{1}{2}}} \quad (5)$$

Where $\phi(\theta) = \frac{1}{4}(1-cos\theta)^2(2+cos\theta)$ is function of wetting angle θ.

1.2. Definition and computational methods of Roughness and Overall trend

We extracted the sediment and boundary contour lines from raw calculation data, and then fit the boundary contour lines to obtain a smooth curve that can represent the overall structure of the sediment. According to the obtained curve, the standard deviation is calculated to characterize the local roughness R_a , and the overall trend ε characterizing the overall structure can also be calculated by equation(6), the schematic diagram of the fitting curve and outline is shown in **Error! Reference source not found.**. The morphology was characterized by the same method by using different conditions, and the results are shown in SI section 0.

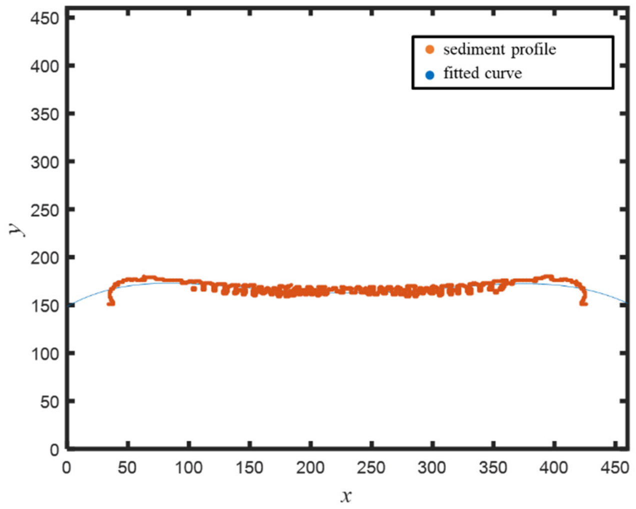

Figure S1. the outline of precipitation and its fit curve.

$$\varepsilon = \frac{y_{left} - y_{mid}}{\min(y_{left}, y_{mid})} \tag{6}$$

where y is the y-axis coordinate of the sediment profile fitting curve, and for the bimodal curve, there are three real extreme values in the calculation area point, the subscripts *left* and *mid* represent the two extreme values in the left and middle of the fitted curve, respectively. For the unimodal curve, there is only one extreme point, so the subscript *left* represents the leftmost value of the actual sediment. This value represents the overall trend of the sediment morphology. The larger the absolute value, the greater the fluctuation and the negative value indicates that the sediment is a single peak structure.

2. Detailed parameter table

2.1. Physical and Chemical Parameters

Table S1. Physical and Chemical Parameters.

Parameter name	symbol	unit	value	Parameter name	symbol	unit	value
Scale Factor of time	C_t	s	1.99E-08	*Molar mass of silicon*	m_1	$kg \cdot mol^{-1}$	0.028
Scale Factor of length	C_L	m	8.70E-07	*Density of copper*	ρ_2	$kg \cdot m^{-3}$	8960
Scale Factor of density	C_ρ	$kg \cdot m^{-3}$	199.82	*Thermal conductivity of copper*	k_2	$W \cdot m^{-1} \cdot K^{-1}$	401
Scale Factor of temperature	C_T	K	28.815	*Specific heat capacity of copper*	c_2	$J \cdot kg^{-1} \cdot K^{-1}$	390
Scale Factor of amount of substance	C_{mol}	mol	1.00E-06	*Molar mass of copper*	m_2	$kg \cdot mol^{-1}$	0.064
Simulation area length	L	m	0.0004	*Diffusion coefficient*	D	$m^2 \cdot s^{-1}$	1.00E-06
Gas constant	R	$J \cdot mol^{-1} \cdot K^{-1}$	8.314	*Homogeneous Reaction Activation Energy*	E_{a1}	$J \cdot mol^{-1}$	80000
laser pulse repetition rate	*Rep*	Hz	50000	*Chemical reaction rate constant*	a_1		3.80E+09
Laser pulse width	w	s	1.00E-07	*The saturation concentration of copper in water*	C_{s2}	$mol \cdot m^{-3}$	8.00E-16
Laser peak power	P	$W \cdot m^{-3}$	1.11E+09	*Growth barrier*	E_{a2}	$J \cdot mol^{-1}$	70000
Density of water	ρ_0	$kg \cdot m^{-3}$	999.1	*growth constant*	a_2		1.00E+09
Thermal conductivity of water	k_0	$kg \cdot m^{-3}$	0.62	*Contact angle*	θ	rad	0.15
Thermal expansion coefficient of water	β_0	——	0.00289	*diffusion barrier*	E_u	$J \cdot mol^{-1}$	80000
Specific heat capacity of water	c_0	$J \cdot kg^{-1} \cdot K^{-1}$	4189	*Molecular Collision Frequency*	ω	Hz	2.00E+13

Molar mass of water	m_0	$kg \cdot mol^{-1}$	0.018	*Molecular mean free path*	l_0	m	3.00E-10
Dynamic viscosity of water	μ	$N \cdot s \cdot m^{-2}$	0.00113757	*molecular volume*	v_0	m^3	1.41E-29
Density of silicon	ρ_1	$kg \cdot m^{-3}$	2330	*Copper-Silicon-Water Surface Energy*	σ	$J \cdot m^{-2}$	0.1
Thermal conductivity of silicon	k_1	$W \cdot m^{-1} \cdot K^{-1}$	149	*Laser penetration depth*	l_p		50
Specific heat capacity of silicon	c_1	$J \cdot kg^{-1} \cdot K^{-1}$	706.75	*Initial concentration of reactants A or B*	C_0	$J \cdot mol^{-1}$	10000

2.2. Working condition parameters

Table S2. condition name and its detailed parameters.

Condition number	D	E_a	E_b	θ	E_d	σ	C0
1	1.00E-06	60000	70000	0.15	80000	0.1	1000
2	1.00E-06	80000	70000	0.15	80000	0.1	1000
3	1.00E-06	70000	70000	0.15	80000	0.1	1000
4	1.00E-06	70000	70000	0.15	80000	0.15	1000
5	1.00E-06	70000	70000	0.15	80000	0.2	1000
6	1.00E-06	70000	70000	0.15	80000	0.3	1000
7	1.00E-06	70000	70000	0.15	80000	0.4	1000
8	1.00E-06	70000	70000	0.15	80000	0.5	1000
9	1.00E-06	70000	70000	0.15	80000	0.1	500
10	1.00E-06	70000	70000	0.15	10000	0.1	500
11	1.00E-06	70000	70000	0.15	100000	0.1	500
12	1.00E-06	70000	70000	0.15	60000	0.1	500
13	1.00E-06	70000	70000	0.15	50000	0.1	500
14	1.00E-06	70000	70000	0.15	80000	0.1	100
15	1.00E-06	70000	70000	0.15	80000	0.1	50
16	1.00E-06	70000	70000	0.15	80000	0.1	200
17	1.00E-06	70000	70000	0.15	80000	0.1	300
18	1.00E-06	70000	70000	0.05	80000	0.1	500
19	1.00E-06	70000	70000	0.02	80000	0.1	500
20	1.00E-06	70000	70000	0.2	80000	0.1	500
21	1.00E-06	70000	70000	0.25	80000	0.1	500
22	1.00E-06	100000	70000	0.15	80000	0.1	1000
23	1.00E-06	120000	70000	0.15	80000	0.1	1000
24	1.00E-06	70000	100000	0.1	80000	0.1	500
25	1.00E-06	70000	120000	0.1	80000	0.1	500
26	1.00E-06	70000	140000	0.1	80000	0.1	500
27	1.00E-06	70000	60000	0.1	80000	0.1	500
28	1.00E-06	70000	40000	0.1	80000	0.1	500
29	1.00E-06	70000	20000	0.1	80000	0.1	500
30	3.00E-08	80000	70000	0.15	80000	0.1	1000
31	1.00E-04	120000	70000	0.15	80000	0.1	1000
32	5.00E-06	70000	70000	0.1	80000	0.1	500
33	1.00E-05	70000	70000	0.1	80000	0.1	500
34	5.00E-05	70000	70000	0.1	80000	0.1	500
35	1.00E-07	70000	70000	0.1	80000	0.1	500

36	1.00E-08	70000	70000	0.1	80000	0.1	500
37	5.00E-09	70000	70000	0.1	80000	0.1	500

3. Simulation results under different working conditions

3.1.1. Roughness, average deposition rate and fluctuation of overall structure of sediment

Table S3. Sediment morphology parameter values under different conditions.

Condition number	Time steps	R_a	ε	R_G	Condition number	Time steps	R_a	ε	R_G
1	100000	4997.12	-0.03	0.52841	19	100000	5.48	0.17	0.00777
	200000	1666.79	1.82			200000	10.22	0.34	
	300000	1521.05	2.22			300000	14.02	0.54	
	400000	1480.72	2.31			400000	25.01	0.62	
	500000	1461.19	2.30			500000	36.76	0.73	
	600000	1449.23	2.35			600000	48.47	0.91	
	700000	1451.74	2.25			700000	62.70	1.03	
	800000	1440.82	2.33			800000	79.91	1.24	
	900000	1454.85	2.14			900000	106.89	1.43	
2	100000	9.34	0.22	0.01565	20	100000	5.48	0.17	0.00777
	200000	30.03	0.35			200000	10.22	0.34	
	300000	57.86	0.52			300000	14.05	0.54	
	400000	96.07	0.66			400000	24.94	0.63	
	500000	147.82	0.76			500000	36.81	0.74	
	600000	195.93	0.88			600000	48.37	0.92	
	700000	256.61	1.01			700000	62.86	1.03	
	800000	330.57	1.10			800000	81.52	1.25	
	900000	415.35	1.09			900000	108.30	1.44	
3	100000	9.78	0.22	0.01952	21	100000	5.48	0.17	0.00777
	200000	28.94	0.38			200000	10.22	0.34	
	300000	60.35	0.58			300000	14.02	0.54	
	400000	107.24	0.80			400000	24.91	0.63	
	500000	161.87	1.04			500000	36.83	0.73	
	600000	236.02	1.31			600000	48.31	0.92	
	700000	318.44	1.55			700000	63.01	1.04	
	800000	450.96	1.89			800000	81.41	1.24	
	900000	701.68	2.69			900000	107.99	1.45	
4	100000	9.79	0.22	0.01961	22	100000	9.48	0.19	0.01103
	200000	29.11	0.36			200000	23.05	0.34	
	300000	60.08	0.57			300000	52.36	0.55	
	400000	107.14	0.79			400000	94.27	0.69	
	500000	161.43	1.03			500000	148.44	0.76	
	600000	234.64	1.28			600000	214.90	0.81	
	700000	317.21	1.53			700000	288.88	0.83	
	800000	452.12	1.91			800000	376.39	0.83	
	900000	701.93	2.77			900000	473.94	0.89	
5	100000	9.78	0.22	0.01961	23	100000	4.99	0.04	0.00914
	200000	28.97	0.37			200000	13.67	0.15	

	300000	60.01	0.57			300000	29.50	0.29	
	400000	107.83	0.78			400000	58.82	0.41	
	500000	161.52	1.02			500000	104.09	0.49	
	600000	234.80	1.29			600000	161.12	0.57	
	700000	317.28	1.49			700000	234.92	0.63	
	800000	454.35	1.88			800000	327.02	0.72	
	900000	703.43	2.68			900000	440.70	0.75	
6	100000	9.82	0.22	0.01963	24	100000	3.05	0.07	0.00548
	200000	28.92	0.38			200000	5.32	0.10	
	300000	59.91	0.57			300000	7.62	0.28	
	400000	108.10	0.77			400000	12.51	0.28	
	500000	161.72	1.02			500000	16.31	0.45	
	600000	234.58	1.29			600000	19.77	0.56	
	700000	318.46	1.52			700000	24.87	0.65	
	800000	452.58	1.93			800000	32.02	0.74	
	900000	702.27	2.79			900000	39.60	0.87	
7	100000	9.79	0.22	0.01968	25	100000	3.04	-1.64	0.00416
	200000	28.95	0.37			200000	4.00	-4.18	
	300000	59.80	0.55			300000	4.17	-4.79	
	400000	107.51	0.75			400000	4.98	0.05	
	500000	161.51	0.99			500000	10.10	0.06	
	600000	234.05	1.29			600000	19.48	0.12	
	700000	313.78	1.54			700000	35.26	0.07	
	800000	449.15	1.98			800000	59.19	0.02	
	900000	696.57	2.80			900000	86.81	-0.94	
8	100000	9.79	0.22	0.01968	27	100000	5.83	0.16	0.00825
	200000	29.10	0.36			200000	13.56	0.32	
	300000	59.50	0.54			300000	22.67	0.60	
	400000	106.82	0.74			400000	35.19	0.79	
	500000	160.81	0.98			500000	50.66	0.96	
	600000	234.26	1.23			600000	68.00	1.18	
	700000	316.18	1.48			700000	90.59	1.38	
	800000	453.50	1.90			800000	120.71	1.60	
	900000	705.43	2.74			900000	148.75	1.84	
9	100000	5.48	0.17	0.01968	28	100000	1985.32	-0.42	2.17482
	200000	10.22	0.34			200000	2213.93	-0.42	
	300000	14.06	0.54			300000	2438.85	-0.42	
	400000	24.95	0.63			400000	2433.63	-0.46	
	500000	36.83	0.73			500000	2446.49	-0.47	
	600000	48.37	0.92			600000	2448.14	-0.49	
	700000	62.86	1.04			700000	2453.45	-0.51	
	800000	80.35	1.25			800000	2453.26	-0.51	
	900000	106.78	1.43			900000	2460.62	-0.51	
11	100000	5.55	0.18	0.01968	30	100000	0.00	0.00	0.00123
	200000	10.34	0.34			200000	9.42	0.26	
	300000	14.25	0.55			300000	10.33	0.41	
	400000	24.97	0.61			400000	18.10	0.37	
	500000	36.62	0.72			500000	20.30	0.35	

	600000	48.84	0.90			600000	21.40	0.25	
	700000	61.55	1.05			700000	29.08	0.21	
	800000	80.83	1.21			800000	31.85	0.22	
	900000	106.01	1.36			900000	35.07	0.19	
12	100000	5.48	0.17	0.01968	31	100000	17.92	0.09	0.26951
	200000	10.22	0.34			200000	113.80	0.16	
	300000	14.02	0.54			300000	752.23	-2.98	
	400000	25.01	0.62			400000	1749.81	-2.02	
	500000	36.76	0.73			500000	3458.01	0.04	
	600000	48.44	0.92			600000	2360.50	8.25	
	700000	62.76	1.03			700000	2263.90	9.28	
	800000	79.93	1.24			800000	2258.48	9.45	
	900000	106.97	1.43			900000	2226.72	9.59	
13	100000	5.48	0.17	0.01968	32	100000	9.34	0.42	0.26951
	200000	10.22	0.34			200000	27.60	0.79	
	300000	14.02	0.54			300000	58.41	1.09	
	400000	24.97	0.63			400000	112.36	1.30	
	500000	36.76	0.73			500000	171.14	1.56	
	600000	48.44	0.92			600000	263.33	1.90	
	700000	62.76	1.03			700000	347.20	2.04	
	800000	79.93	1.24			800000	445.32	2.06	
	900000	106.97	1.43			900000	562.56	1.98	
14	200000	0.00	0.00	0.00108	33	100000	12.13	0.59	0.03068
	300000	0.00	0.00			200000	43.67	1.06	
	400000	5.56	0.70			300000	109.00	1.29	
	500000	4.69	1.60			400000	195.40	1.59	
	600000	5.26	0.56			500000	321.19	1.82	
	700000	6.95	0.59			600000	457.79	2.04	
	800000	6.19	0.76			700000	648.47	2.11	
	900000	7.88	1.38			800000	899.67	1.92	
15	400000	0.00	0.00	0.00046		900000	1185.95	1.70	
	500000	0.00	0.00		34	100000	24.30	1.06	0.08892
	600000	0.00	0.00			200000	106.32	1.13	
16	100000	0.00	0.00	0.00244		300000	277.92	1.15	
	200000	2.90	0.09			400000	495.48	1.46	
	300000	6.42	0.66			500000	837.04	1.71	
	400000	8.32	0.39		35	100000	0.00	0.00	
	500000	9.96	0.83			200000	0.00	0.00	
	600000	12.08	0.82			300000	7.81	0.48	
	700000	14.69	0.97			400000	7.62	0.47	
	800000	16.06	1.23			500000	10.53	1.16	
	900000	20.34	1.13		36	600000	0.00	0.00	0.00036
17	100000	5.48	0.17	0.00777		700000	0.00	0.00	
	200000	10.22	0.34			800000	0.00	0.00	
	300000	14.06	0.54			900000	0.00	0.00	
	400000	24.97	0.63		38	100000	9.34	0.22	
	500000	36.81	0.74			200000	30.04	0.35	
	600000	48.42	0.91			300000	58.17	0.51	

	700000	62.91	1.03			400000	96.22	0.65	
	800000	80.35	1.25			500000	147.31	0.76	
	900000	107.10	1.43			600000	194.36	0.89	
18	100000	5.48	0.17	0.00777		700000	257.96	0.98	
	200000	10.22	0.34			800000	333.47	1.11	
	300000	14.02	0.54			900000	418.94	1.11	
	400000	24.97	0.63						
	500000	36.92	0.73						
	600000	48.42	0.91						
	700000	62.80	1.04						
	800000	79.97	1.24						
	900000	106.89	1.42						

3.1.2. Cloud map under different conditions

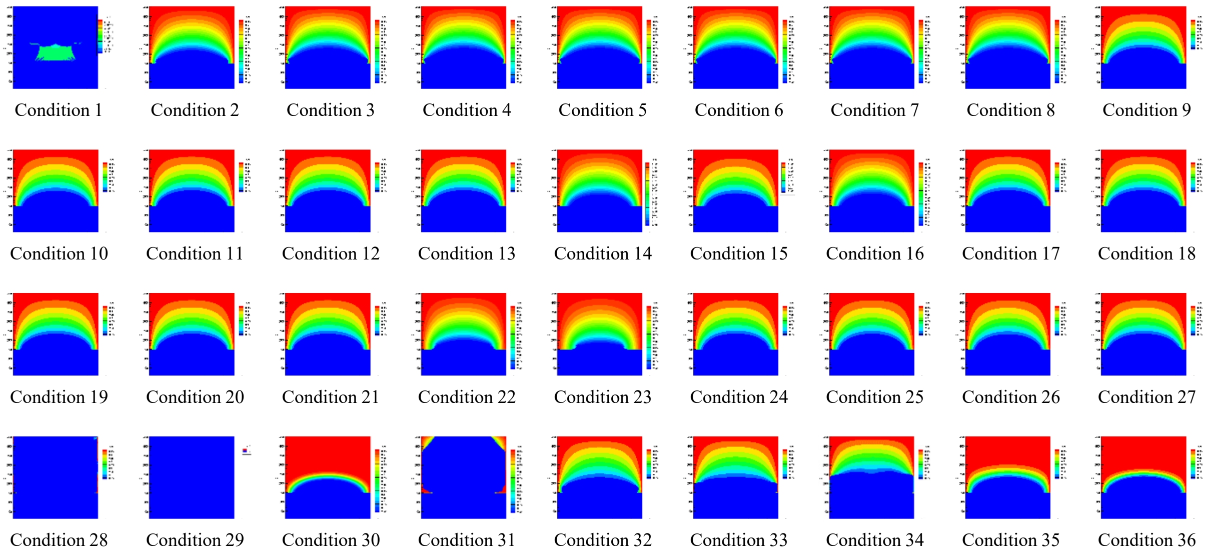

Figure S2. The concentration distribution of A(aq) under different conditions at time step of 500,000

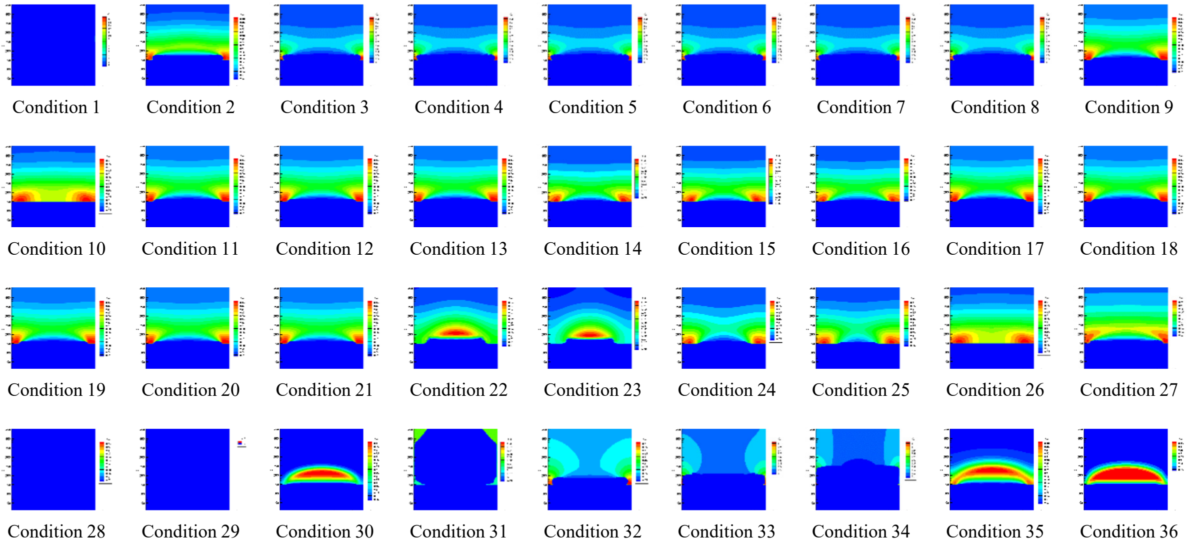

Figure S3. The concentration distribution of C(aq) under different conditions at time step of 500,000.

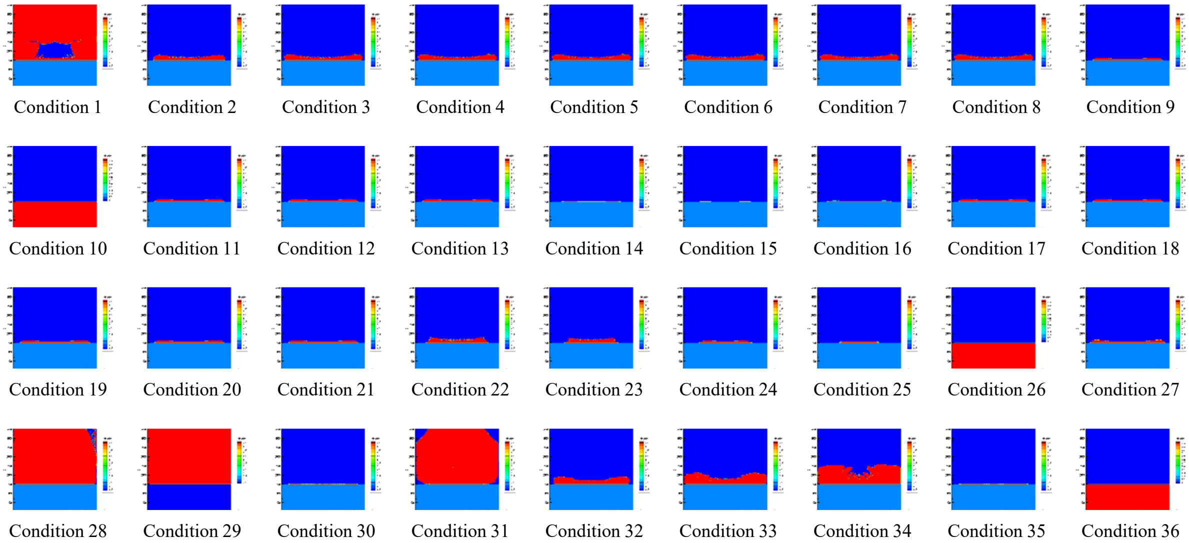

Figure S4. the diagram of Morphology under different conditions at the time step of 500,000.

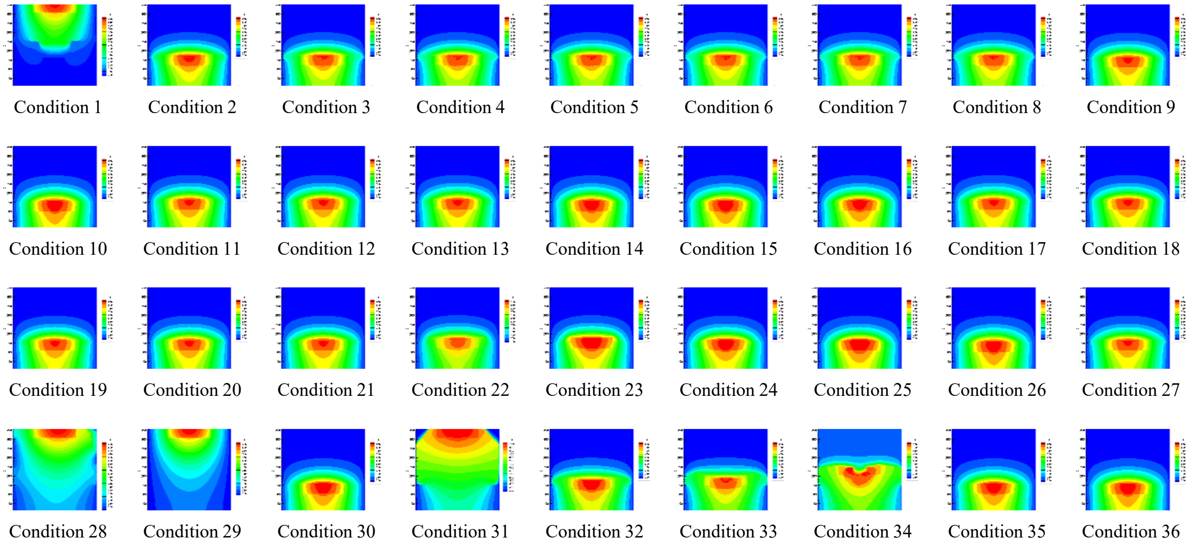

Figure S5. The temperature distribution under different conditions at the time step of 500,000.

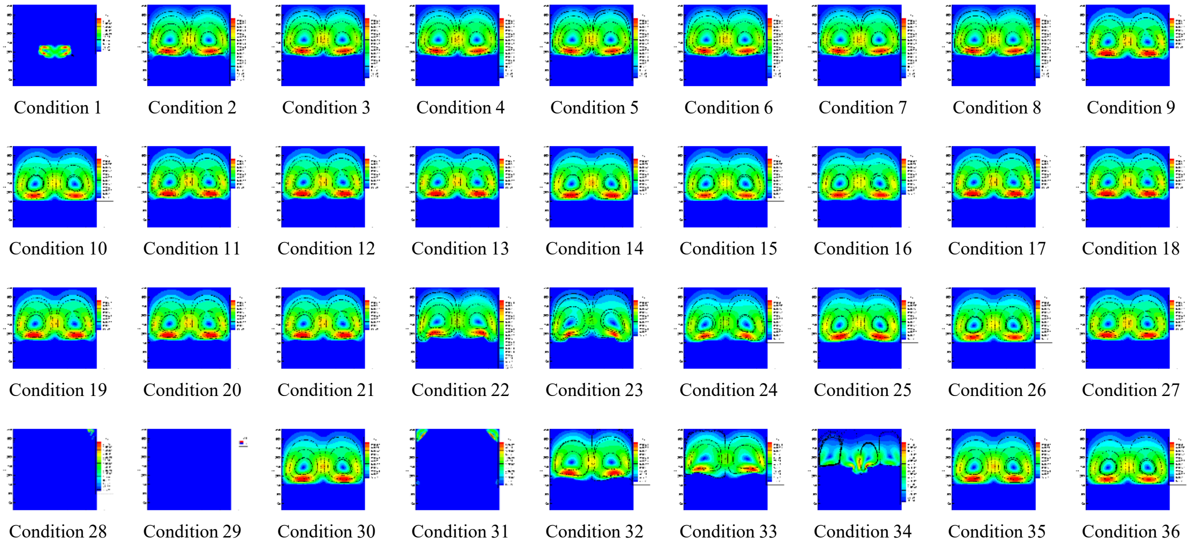

Figure S6. Streamlines and Velocity Contours under different conditions at the time step of 500,000.
